# Integrative Analyses of Metabolites and Transcriptome Reveal the Metabolic Pattern of Glucosinolates in Potherb Mustard (*Brassica juncea* var. *multiceps*)

**DOI:** 10.3390/plants13172481

**Published:** 2024-09-05

**Authors:** Jie Wang, Shunhao Yu, Xiliang Ren, Yating Wang, Zhongrui Wang, Qiufeng Meng, Yunping Huang, Yuhong Wang

**Affiliations:** 1 Ningbo Academy of Agricultural Sciences, Ningbo 315000, China; jieer881018@163.com (J.W.); xl_ren@126.com (X.R.); qfmeng@163.com (Q.M.); 2 Ningbo Key Laboratory of Characteristic Horticultural Crops in Quality Adjustment and Resistance Breeding, Ningbo 315000, China; 3 Key Laboratory of Horticultural Plant Growth, Development and Quality Improvement, Ministry of Agriculture, Department of Horticulture, Zhejiang University, Hangzhou 310058, China; yushunhao@zju.edu.cn (S.Y.); wang_yt@zju.edu.cn (Y.W.); wangzhongrui0-o@zju.edu.cn (Z.W.)

**Keywords:** potherb mustard, varieties, glucosinolate, hydrolysis

## Abstract

Potherb mustard (*Brassica juncea* var. *multiceps*) is one of the most commonly consumed leafy vegetable mustards, either fresh or in pickled form. It is rich in glucosinolates, whose hydrolyzed products confer potherb mustard’s distinctive flavor and chemopreventive properties. In this study, the composition and content of glucosinolates, as well as the hydrolysis pattern of sinigrin were investigated in potherb mustard leaves of different varieties. Variations in the glucosinolate profile and accumulation were observed among the potherb mustard varieties studied, with sinigrin being the predominant one in all varieties, accounting for 81.55% to 97.27%. Sinigrin tended to be hydrolyzed to isothiocyanate (ITC) rather than epithionitrile (EPN) in potherb mustard, while 3-butenyl nitrile (SIN-NIT) could be hardly detected. Transcriptome analysis revealed a higher expression level of numerous genes involved in aliphatic glucosinolate biosynthesis in X11 compared to X57, corresponding to the higher aliphatic glucosinolate accumulation in X11 (91.07 µmol/g) and lower level in X57 (25.38 µmol/g). ESM1 is known to repress nitrile formation and favor isothiocyanate production during glucosinolate hydrolysis. In this study, all four *ESM1s* showed a higher expression level in X11 compared to X57, which may determine the hydrolysis pattern of sinigrin in potherb mustard. Altogether, our findings shed light on the glucosinolate metabolic pattern in potherb mustard, which will also facilitate the engineering of metabolic pathways at key checkpoints to enhance bioactive compounds for tailored flavor or pharmaceutical needs.

## 1. Introduction

Potherb mustard (*Brassica juncea* var. *multiceps*) is a leafy vegetable belonging to the *Brassica* crops. With a long history of cultivation in China, it stands as one of the most commonly consumed vegetables, either in fresh leafy greens or in pickled form. Potherb mustard is rich in glucosinolates, whose hydrolysis products constitute a significant portion of the volatile constituents in pickled products, imparting potherb mustard with its distinctive flavor [1].

Glucosinolates are a group of nitrogen- and sulfur-containing plant secondary metabolites, which mainly occur in *Brassicaceae* plants and are abundant in *Brassica* vegetables. Numerous glucosinolates have been characterized from plants and they can be classified into aliphatic glucosinolates, indolic glucosinolates, and benzenic glucosinolates based on their different amino acid precursors [2]. Specifically, aliphatic glucosinolates are those derived from methionine, alanine, leucine, isoleucine, and valine; indolic glucosinolates are those derived from tryptophan; and benzenic glucosinolates are those derived from phenylalanine and tyrosine. In general, glucosinolates that remain unhydrolyzed are considered to have no biological activity. When tissue is disrupted, the endogenous enzymes termed myrosinases (thioglucoside glucohydrolases, TGGs) come into contact with glucosinolates and hydrolyze them into unstable aglucone, which undergoes spontaneous rearrangement and generates isothiocyanates (ITCs) [3]. In addition, thiocyanates, epithionitriles (EPNs), and nitriles (NITs) can also be generated. The type of degradation product is determined by factors such as glucosinolate structure, pH value, ferrous ion concentration, and the presence of specific proteins. These proteins include the thiocyanate-forming proteins (TFPs), epithiospecifiers (ESPs), nitrile specifier proteins (NSPs), and EPITHIOSPECIFIER MODIFIER1 (ESM1) [4,5]. Among them, nitriles can be formed in the presence of NSPs, while epithionitriles are catalyzed by ESP. ESM1 represses nitrile formation and favors isothiocyanate production. These hydrolysis products, particularly ITCs, contribute to the well-known bioactivity of glucosinolates in plant defense, vegetable flavor, and human health promotion [6,7].

The pattern of glucosinolate accumulation and degradation differs dramatically among different species and varieties. Numerous studies on glucosinolate accumulation patterns in vegetable and oilseed *B. juncea* have emerged in recent years, with sinigrin and gluconapin being identified as the predominant glucosinolate profiles [8,9,10]. However, for vegetable *B*. *juncea*, reports primarily focus on stem mustard [9,10,11]. Since the hydrolytic products are the main biologically active form of glucosinolates, their diverse pattern and underlying mechanisms deserve more attention. The profile and concentration of glucosinolate breakdown products in seedlings of Chinese kale (*B*. *oleracea* var. *alboglabra*) and broccoli (*B*. *oleracea* var. *italica*) have been detected, and the expression of *BoESP*s has been reported to control the pattern of glucosinolate breakdown products in Chinese kale seedlings, while *BoESP*s and *BoNSP*s dominate that in broccoli seedlings [12,13]. Research on the glucosinolate hydrolysis pattern in mustard, particularly leafy mustard, is scarcer. Púčiková et al. (2023) and Di et al. (2023) determined the profile and content of glucosinolate breakdown products in leafy mustard Red Giant [14] and Zhuchang [15], respectively. However, neither of them referred to the molecular mechanism. In this study, we present the composition and contents of glucosinolates in potherb mustard of different varieties. Additionally, we investigate the types of glucosinolate breakdown products among different potherb mustard varieties and identify the underlying mechanisms through whole-genome transcriptome analysis.

## 2. Results and Discussion

### 2.1. Glucosinolate Pattern in Potherb Mustard of Different Varieties

A total of 11 individual glucosinolates were detected in the leaves of 68 potherb mustard varieties tested (X1~X68). These included 6 aliphatic glucosinolates (glucoiberverin, glucoiberin, sinigrin, gluconapin, progoitrin, glucobrassicanapin), 4 indolic glucosinolates (glucobrassicin, neoglucobrassicin, 4-hydroxy glucobrassicin, 4-methoxy glucobrassicin), and 1 benzenic glucosinolate (gluconasturtiin) (Table S1 and Figure S1). Among these, sinigrin, gluconapin, progoitrin, glucobrassicin, neoglucobrassicin, and 4-methoxy glucobrassicin can be detected in all 68 varieties. Only 15 varieties have glucobrassicanapin, and 23 varieties have gluconasturtiin. In addition, their contents were relatively low, ranging from 0.02 to 0.42 μmol/g for glucobrassicanapin and from 0.02 to 0.70 μmol/g for gluconasturtiin. This profile is different from reports in other *B. juncea* vegetables. In tuber mustard (*B. juncea* var. *tumida*), 3 kinds of aliphatic glucosinolates were detected [10]. Moreover, only a total of 3 glucosinolates could be detected in leafy vegetable giant red mustard (*B. juncea* ssp. *rugosa* cv. Red Giant) [14]. Since glucosinolates have diverse and important ecological functions, their profiles are shaped by natural and artificial selection [16]. Furthermore, these differences might also result from limitations in the detection capabilities of different machines, which could omit profiles with trace amounts.

**Figure 1:**
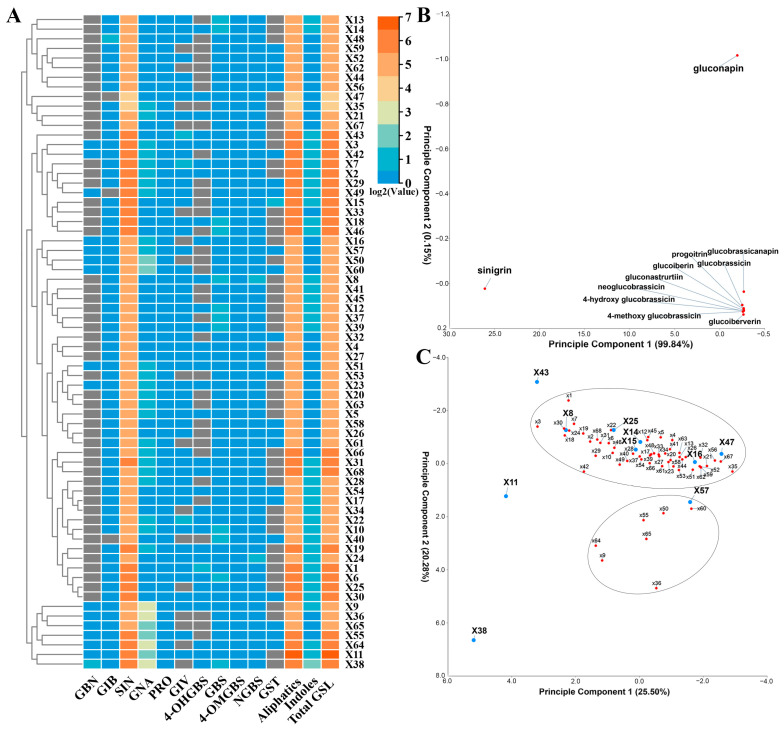
Glucosinolate composition and content in potherb mustard (*Brassica juncea* var. *multiceps*) of different varieties (**A**), as well as PCA of different glucosinolate profiles (**B**) and different varieties (**C**) based on the contents presented in Table S2. Values represent the means of three to four replicates. The heatmap was plotted using TBtools-II (v2.097) with Log2-transformed FPKM values. GBN, glucobrassicanapin; GIB, glucoiberin; SIN, sinigrin; GNA, gluconapin; PRO, progoitrin; GIV, glucoiberverin; 4-OHGBS, 4-hydroxyglucobrassicin; GBS, glucobrassicin; 4-OMGBS, 4-methoxyglucobrassicin; NGBS, neoglucobrassicin; GST, gluconasturtiin; Aliphatics, aliphatic glucosinolates; Indoles, indolic glucosinolates; Total GSL, total glucosinolates.

There were significant differences in the total glucosinolate content among different varieties of potherb mustard, ranging from 17.46 μmol/g in X47 to 91.85 μmol/g in X11. However, aliphatic glucosinolates predominated in all varieties, comprising 88.67% to 99.20% of the total content. In line with previous reports in *B. juncea* crops [10,17,18,19], sinigrin is the most abundant aliphatic profile in all varieties, accounting for 82.76% to 99.36%, followed by gluconapin (Figure 1A and Table S2). Progoitrin occurred at low levels varying from 0.02 μmol/g to 0.12 μmol/g. Consistently, the PCA revealed that sinigrin exhibited high variance from other types of glucosinolates in PC1, explaining 99.84% of the variance. Gluconapin followed in PC2, explaining 0.15% (Figure 1B). This confirmed that potherb mustard evolved the capacity for sinigrin super-accumulation. Interestingly, among 68 varieties, X47 has the lowest contents of sinigrin and progoitrin, and X11 harbors the richest ones. As sinigrin- and progoitrin-derived compounds are related to the pungency and bitterness in mustard [20,21], X47 may possess a lighter taste, making it a potential candidate for breeding fresh leafy greens. Meanwhile, X11 exhibits a stronger taste, making it more suitable for consumption after processing [10].

In potherb mustard, the content of indolic glucosinolates varied from 0.07 μmol/g in X35 to 1.55 μmol/g in X8, accounting for 1.31% of total glucosinolates on average (Figure 1A and Table S2). The relatively low content of indolic glucosinolates may be due to the fact that the leaf materials were harvested at a mature stage. Frazie et al. (2017) have determined indolic glucosinolate concentration in the leaves of 11 mustard cultivars at baby leaf and mature stages and found that indolic glucosinolate concentration decreases along with leaf age [21]. Furthermore, *Brassicaceae* plants naturally possess a baseline level of indolic glucosinolates to adapt and survive in changing environments, as indolic glucosinolates play an important role in plant immune response [22]. Mustard crops are always selected for their distinctive flavor or as a condiment. Thus, aliphatic glucosinolates, which contribute more to the flavor, have evolved to be highly expressed, while indolic ones are kept at the baseline.

We also carried out PCA to identify batches of varieties having similar glucosinolate patterns (Figure 1C). In comparison to the PCA of glucosinolates (Figure 1B), the PC1 (20.50%) and PC2 (20.28%) accounted for a lower variance when applied to varieties (Figure 1C). It resulted in 5 groups: X48, X11, X43, and other two clusters discriminated by two circles in Figure 1C.

### 2.2. The Pattern of Sinigrin Breakdown Products in Potherb Mustard among Different Varieties

**Figure 2:**
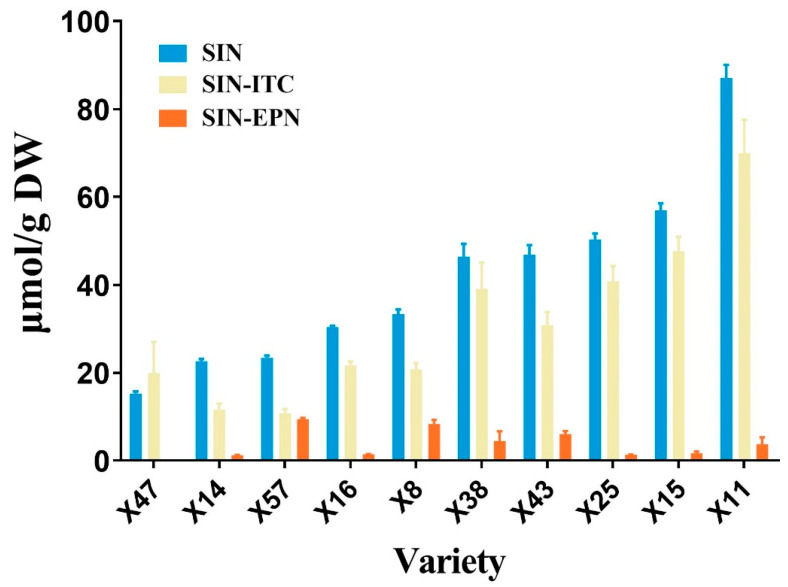
Sinigrin breakdown products in different varieties of potherb mustard leaves. SIN, sinigrin; SIN-ITC, 2-propenyl isothiocyanate; SIN-EPN, 3,4-epithiobutyl nitrile.

Since sinigrin is the predominant profile and its breakdown products are the main contributors to the special flavor of potherb mustard [1], we further investigated the hydrolysis pattern of sinigrin in potherb mustard leaf. Different varieties of potherb mustard were selected according to the cluster analysis and PCA (Figure 1), so as to cover as many varieties as possible. Results showed that potherb mustard predominantly released 2-propenyl ITC (SIN-ITC) from sinigrin, accounting for 53.35% in X57 to 100% in X47. This was followed by 3,4-epithiobutyl nitrile (SIN-EPN), which ranged from 0% in X47 to 28.73% in X8. We could hardly detect 3-butenyl nitrile (SIN-NIT) (Figure 2). It is similar to the previous findings in another leafy mustard (*B. juncea* ssp. *rugosa* cv. Red Giant) and the *B. juncea* leaf [14,21]. However, SIN-NIT showed a higher concentration than SIN-EPN in the seeds of potherb mustard, although SIN-ITC is still the most abundant breakdown product [23]. Púčiková et al. (2023) reported that old leaves released more ITCs and fewer EPNs than young leaves in leafy mustard [14]. In addition, in Chinese kale, the proportion of ITCs in total glucosinolate breakdown products is the richest in seeds, but not in seedlings [12]. These suggest that the hydrolysis pattern of glucosinolate may be associated with the plant’s developmental stage. In addition, the content of ITC products is comparable to that of NITs in tuber mustard [9], and is comparable to that of EPNs in *B. juncea* var. *rugosa* cv. ZC-2 [15], indicating the diversity of hydrolysis patterns of glucosinolate among species. It has been reported that the odor of SIN-ITC is 300-fold stronger than that of SIN-EPN, and SIN-ITC also has a more powerful chemopreventive effect [24,25]. Therefore, X11 can be a good choice for pungent-flavored mustard, which has a high level of SIN-ITC.

### 2.3. Correlation among Different Glucosinolates and Breakdown Products

**Figure 3:**
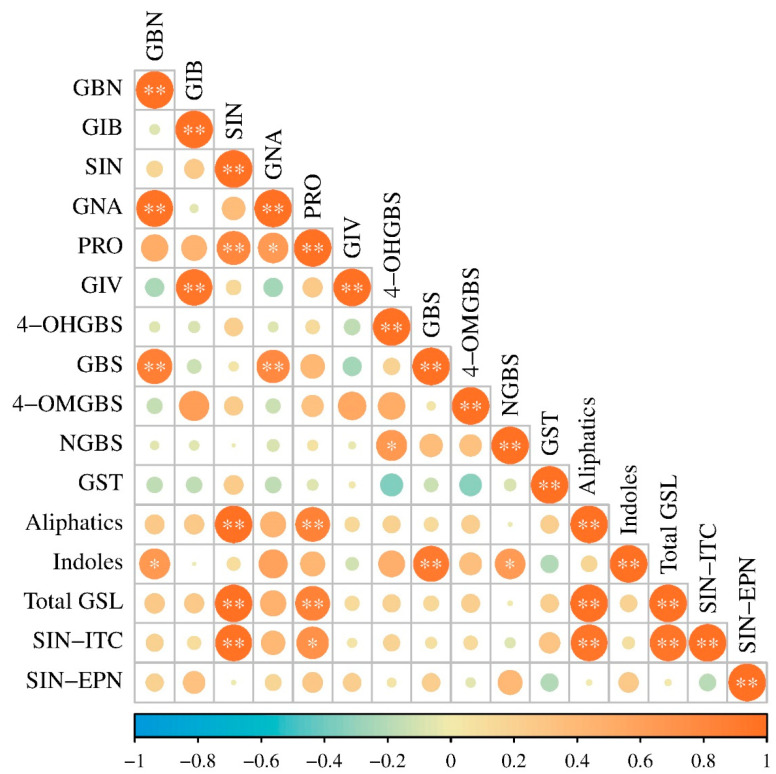
Pearson Correlation Coefficients among different glucosinolates and breakdown products in potherb mustard leaves. * and ** indicate significance at 0.05 and 0.01 probability levels, respectively. The color scale moves from blue (−1.0) to red (1.0) with increased value of Pearson Correlation Coefficients. Heatmap was plotted using https://www.bioinformatics.com.cn (last accessed on 20 February 2024), an online platform for data analysis and visualization. GBN, glucobrassicanapin; GIB, glucoiberin; SIN, sinigrin; GNA, gluconapin; PRO, progoitrin; GIV, glucoiberverin; 4-OHGBS, 4-hydroxyglucobrassicin; GBS, glucobrassicin; 4-OMGBS, 4-methoxyglucobrassicin; NGBS, neoglucobrassicin; GST, gluconasturtiin; Aliphatics, aliphatic glucosinolates; Indoles, indolic glucosinolates; Total GSL, total glucosinolates; SIN-ITC, 2-propenyl isothiocyanate; SIN-EPN, 3,4-epithiobutyl nitrile.

The correlation analysis was conducted to investigate the correlations among different glucosinolates and breakdown products (Figure 3 and Table S2). The results showed that sinigrin had a significant positive correlation with gluconapin. Both compounds contribute to the distinctive flavor and were positively correlated with the total aliphatic glucosinolates (0.996, 0.810; *p* < 0.01). This finding is consistent with studies conducted on stem mustards [10], which are also consumed for their special flavor, suggesting a similar genetic evolution under the pressure of artificial selection. Additionally, two indolic glucosinolates, glucobrassicin and neoglucobrassicin, had significant positive correlations with the total indolic glucosinolates (0.918, *p* < 0.01; 0.649, *p* < 0.05).

In addition, SIN-ITC showed a significant positive correlation with sinigrin (0.962, *p* < 0.01), but SIN-EPN did not. This indicates that different varieties of potherb mustard have the capacity to stably release ITC from sinigrin, but the factors affecting the formation of EPN might vary among different varieties. Overall, the correlation coefficients between sinigrin and aliphatic glucosinolate, total glucosinolates, and SIN-ITC were the top three (0.996, 0.994, 0.962, respectively), indicating its significant contribution to the function of glucosinolates in potherb mustard.

### 2.4. Transcriptome Analysis of Genes in the Glucosinolate-Myrosinase Pathway

To further explore the potential mechanism of glucosinolate biosynthesis and breakdown product pattern in potherb mustard, we performed RNA-seq analysis on X57 and X11 varieties, which exhibited dramatical differences in glucosinolate contents and the proportion of SIN-ITC (Figure 2). A total of 33,559 differentially expressed genes (DEGs) were identified, including 16,807 down-regulated and 16,752 up-regulated genes (Figure S2A). Kyoto Encyclopedia of Genes and Genomes (KEGG) pathway enrichment analysis revealed that many genes were enriched in signaling pathways close to photosynthesis and energy metabolism, while ‘glucosinolate biosynthesis’ was included in the top 20 of down-regulated KEGG pathways (Figure S3).

**Figure 4:**
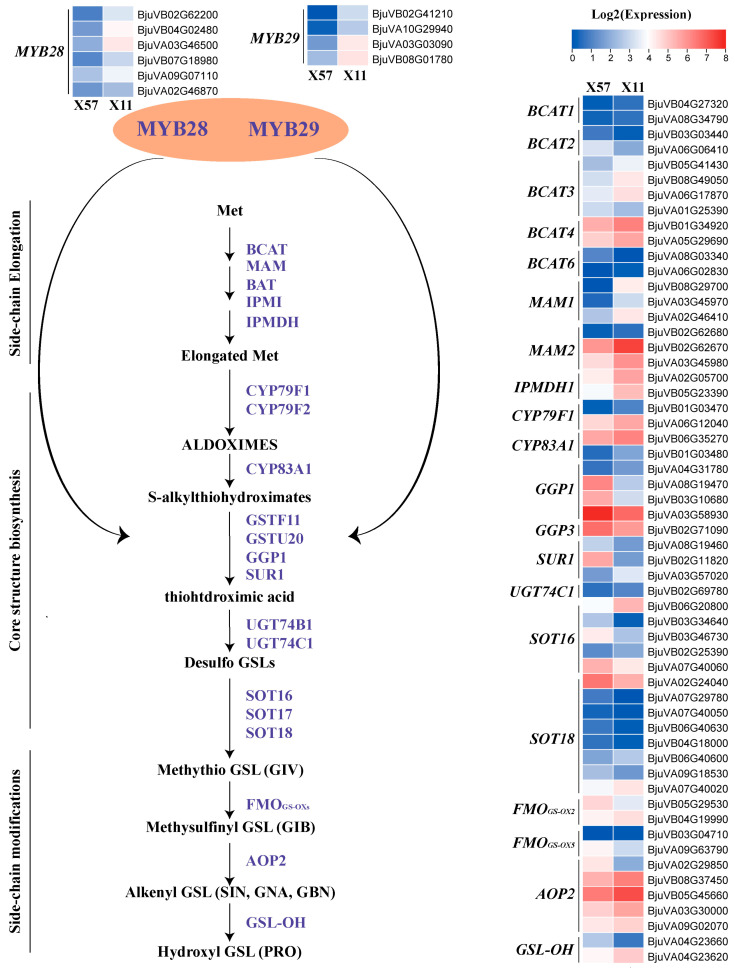
The changes of differentially expressed genes related to glucosinolate biosynthesis in X57 and X11. Values represent the means of three replicates. The expression levels were visualized using TBtools-II (v2.097) with Log2-transformed FPKM values.

Nowadays, the gene inventory of the glucosinolate metabolic pathway has been described widely. Generally, glucosinolate biosynthesis includes three processes: chain elongation, core structure formation, and secondary modification of side chains [26]. Meanwhile, key transcription factors involved in glucosinolate biosynthesis regulation have also been identified, including three R2R3-MYB transcription factors (MYB28, MYB29, and MYB76) that play important roles in aliphatic glucosinolate biosynthesis [27]. We closely examined the DEGs involved in glucosinolate metabolism, identifying 147 down-regulated and 154 up-regulated genes (Figure S2B). Among them, 152 DEGs were associated with the biosynthesis of glucosinolates, while 60 DEGs were involved in degradation (Figure S4). We further focused on aliphatic glucosinolate biosynthetic genes and key transcription factors for detailed analysis (Figure 4). This included 20 DEGs related to side-chain elongation (*BCAT*s, *MAM*s, *IMDH1*s), 26 DEGs related to core structure biosynthesis (*CYP79F1*s, *CYP83A1*s, *GGP*s, *SUR1*s, *UGT74C1*, *SOT*s), and 11 DEGs related to side-chain modification (*FMO_GS-OX_*s, *AOP2*s, *GSL-OH*s), as well as 10 DEGs related to transcriptional regulation (*MYB28*s, *MYB29*s). Most of these genes exhibited higher expression levels in X11 when compared to X57, which was consistent with the higher glucosinolate accumulation in X11 (Figure 1 and Table S2). Notably, *AOP2*, responsible for the formation of sinigrin from the alkenylation of glucoiberin [28], showed relatively higher expression among the biosynthetic genes, supporting the capacity of potherb mustard to super-accumulate sinigrin.

**Figure 5:**
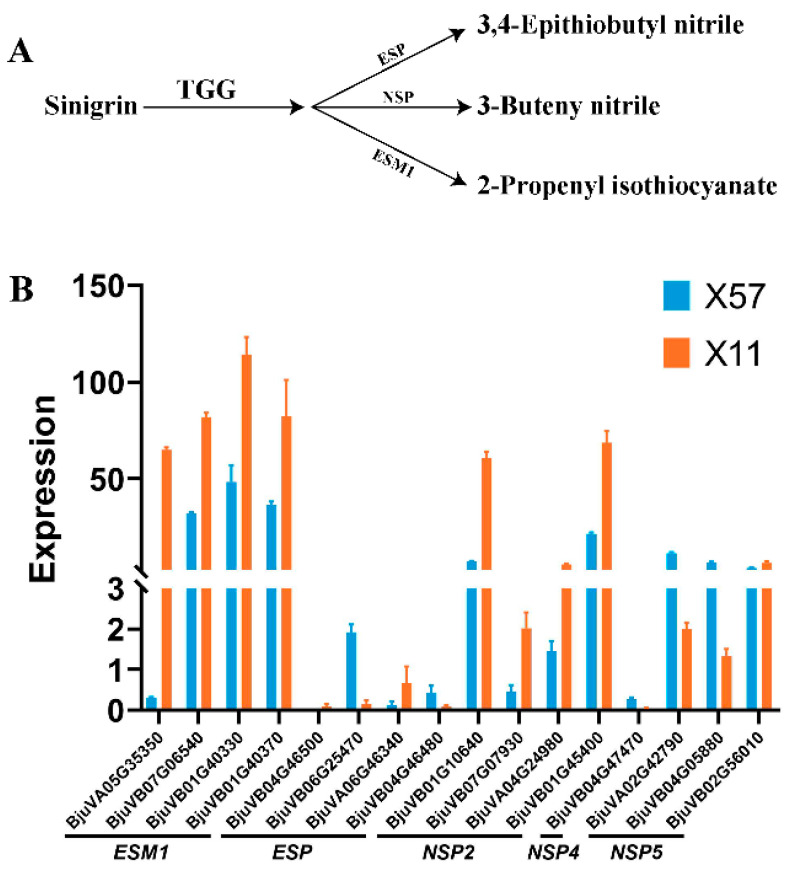
Schematic representation of enzymatic glucosinolate hydrolysis exemplified by sinigrin (**A**), and the gene expression levels of specifier proteins in X57 and X11 (**B**).

In the classical GSL degradation pathway, specifier proteins play an important role in determining the final breakdown products of glucosinolates. Nitriles can be formed in the presence of NSPs [29,30], while epithionitriles are catalyzed by ESP [31,32]. ESM1 represses nitrile formation and favors isothiocyanate production from sinigrin [4]. Studies in broccoli and Chinese kale seedlings revealed that nitriles and epithionitriles are the predominant hydrolytic products, respectively, rather than ITC [12,13]. Their further transcriptome analysis of GSL metabolism-related genes in these species showed that the expression of *BoESP*s and *BoNSP*s might determine the pattern of glucosinolate breakdown products. Here, in our RNA-seq data, a total of 60 DEGs involved in the classical glucosinolate degradation pathway were identified, including 16 specifier protein genes (*ESM1*s, *ESP*s, *NSP*s). As shown in Figure 5B, *ESP*s exhibited relatively lower expression levels compared to *ESM1*s and *NSP*s. Moreover, all four *ESM1*s were highly expressed in X11 compared to X57. Considering SIN-NIT could be hardly detected in this study, the enrichment of *ESM1*s might be the key genes dominating the hydrolysis pattern of sinigrin in potherb mustard.

## 3. Materials and Methods

### 3.1. Plant Materials

Potherb mustard varieties used in this study are the high-generation inbred lines (>F_10_). On 25 October 2022, the seeds were sown in trays in a greenhouse of the Ningbo Academy of Agricultural Sciences (Ningbo, China). Seedlings were grown under controlled conditions (25 °C during the day, 15 °C at night) for 40 days before being transplanted into the field when they had 6–7 true leaves. The field layout was completely randomized, with transplants spaced 35 cm between rows and 30 cm between plants. Standard agricultural practices were followed for watering, fertilization, and pest control.

Mature leaves (free of any insects and mechanical damage) were sampled from five uniform 165-day-old plants, before bolting. For each sampling, three independent replicates were taken. Samples were immediately frozen in liquid nitrogen, transported to the laboratory, and stored at −80 °C until lyophilization. The lyophilized samples were powdered for further analysis.

### 3.2. Determination of Glucosinolates

Glucosinolates were extracted and analyzed as previously described with minor modifications [33]. About 30 mg of powdered samples were added into 2 mL of 90% methanol, vortexed for 3 min, and incubated for 1 h. After centrifugation (3 min, 12,857× *g*), the supernatant was collected and applied to a DEAE-Sephadex A-25 column. The column was washed with 1 mL of 90% methanol and 1 mL of ddH_2_O, and then treated with sulfatase (100 μL of 0.1 mol/L) overnight to convert glucosinolates to desulfoglucosinolates. The desulfoglucosinolates were collected, filtered, and subjected to high-performance liquid chromatography (HPLC) analysis using acetonitrile and water as the mobile phase. The elution procedure included isocratic elution with 1.5% acetonitrile for 5 min, a linear gradient to 20% acetonitrile over 15 min, and then isocratic elution with 20% acetonitrile for 13 min. Absorbance was monitored at 226 nm using Ortho-nitrophenyl-β-D-galactopyranoside as the internal standard. Data were reported as μmol/g DW.

### 3.3. Determination of Glucosinolate Breakdown Products

A modified protocol was used to detect and analyze the degradation products of glucosinolates [33]. A 100 mg sample was dissolved in 1 mL of ddH_2_O together with 0.2 μmol/g of the internal standard benzonitrile solution (Sigma-Aldrich Chemie GmbH, Taufkirchen, Germany, ≥99.9%). The mixture was transferred to a 10 mL centrifuge tube and the residue was washed twice with 1 mL ddH_2_O. The extracts were combined and allowed to stand for 1 h at 25 °C to promote GLS hydrolysis by endogenous myrosinase. Then, 3 mL of dichloromethane (LABOR, Shanghai, China, ≥99.9%) was added. The mixture was vortexed and allowed to stand for 20 min at 25 °C, then centrifuged at 4000× *g* for 10 min. The dichloromethane layer was collected, and the aqueous layer was re-extracted with 3 mL of dichloromethane. Combined extracts were concentrated to 300 μL and analyzed by GC-flame ionization detection (GC-FID) using the Shimadzu GC-2014 instrument (Shimadzu, Kyoto, Japan) with an HP-5ms column (30 m × 0.25 mm × 0.25 μm film). The detection procedure was performed with splitless injection at 250 °C and a hydrogen gas flow rate of 1 mL/min. The column temperature was started at 35 °C for 3 min and then heated to 250 °C for 10 min at a rate of 10 °C/min. The peak area was quantified using benzonitrile as the internal standard, and the final data were expressed as μmol/g DW.

### 3.4. RNA-seq Assay

#### 3.4.1. RNA Isolation and Library Preparation

Total RNA was extracted from frozen samples using the TRIzol reagent (Invitrogen, Carlsbad, CA, USA) according to the manufacturer’s protocol. RNA purity and quantification were evaluated using the NanoDrop 2000 spectrophotometer (Thermo Scientific, Waltham, MA, USA). RNA integrity was assessed using the Agilent 2100 Bioanalyzer (Agilent Technologies, Santa Clara, CA, USA). Then, the libraries were constructed using the VAHTS Universal V6 RNA-seq Library Prep Kit (Vazyme, Nanjing, China) according to the manufacturer’s instructions.

#### 3.4.2. RNA Sequencing and Differentially Expressed Genes Analysis

The transcriptome sequencing and analysis were conducted by OE Biotech Co., Ltd. (Shanghai, China). The libraries were sequenced on an Illumina Novaseq 6000 platform and 150 bp paired-end reads were generated. Raw reads of fastq format were firstly processed using fastp and the low-quality reads were removed to obtain the clean reads. The clean reads were mapped to the reference genome (http://39.100.233.196:82/download_genome/Brassica_Genome_data/Braju_tum_V2.0/Braju_tum_V2.0.fa.gz (accessed on 18 October 2023)) using HISAT22 v2.1.0. FPKM of each gene was calculated and the read counts of each gene were obtained by HTSeq-count. PCA analysis was performed using R (v 3.2.0) to evaluate the biological duplication of samples.

Differential expression analysis was performed using the DESeq2. Q value < 0.05 and foldchange > 1.5 was set as the threshold for significantly differential expression genes (DEGs). Based on the hypergeometric distribution, GO and KEGG pathway enrichment analysis of DEGs were performed to screen the significant enriched term using R (v 3.2.0), respectively.

### 3.5. Statistical Analysis

Principal component analysis (PCA) of glucosinolate or glucosinolate breakdown product profiles was performed by using TBtools software [34]. Correlation analyses were conducted and plotted by https://www.bioinformatics.com.cn (last accessed on 20 February 2024), an online platform for data analysis and visualization [35].

## 4. Conclusions

In summary, the composition and content of glucosinolates varied among the potherb mustard varieties investigated, with sinigrin being the most abundant in all varieties. Furthermore, sinigrin showed a preference for hydrolysis into SIN-ITC in potherb mustard leaves, possibly due to the higher expression of *ESM1*, resulting in a stronger odor of potherb mustard. Among 68 varieties, X11 emerges as a promising candidate for breeding pungent-flavored and chemopreventive mustard, as it possesses the highest levels of sinigrin and progoitrin, as well as SIN-ITC. Collectively, our results provide a valuable resource for advancing molecular and genetic investigations concerning *B. juncea* var. *multiceps*, and offer insights that could facilitate the breeding of customized flavors and functional traits.

## Data Availability

The authors confirm that the data supporting the findings of this study are available within the article and its Supplementary Materials.

## Supplementary Materials

The following supporting information can be downloaded at: https://www.mdpi.com/article/10.3390/plants13172481/s1, Supplementary Table S1: Trivial and chemical name of glucosinolates detected in this study. Supplementary Table S2: The content of GSL in potherb mustard of different varieties (μmol/g DW); Supplementary Figure S1: HPLC chromatograms of desulfoglucosinolates prepared from potherb mustard; Supplementary Figure S2: Volcano plot of all the differentially expressed genes (A) and glucosinolate metabolism-related differentially expressed genes (B) in X57 vs. X11; Supplementary Figure S3: KEGG enrichment analysis of differentially expressed genes in X57 vs. X11; Supplementary Figure S4: The changes of differentially expressed genes related to glucosinolate biosynthesis (A) and degradation (B) in X57 and X11. Values represent the means of three replicates. The expression levels were visualized using TBtools-II (v2.097).

## References

[1] Zhao D., Tang J., Ding X. (2007). Analysis of volatile components during potherb mustard (*Brassica juncea*, Coss.) pickle fermentation using SPME–GC-MS. LWT—Food Sci. Technol..

[2] Wu X., Huang H., Childs H., Wu Y., Yu L., Pehrsson P.R. (2021). Glucosinolates in Brassica vegetables: Characterization and factors That influence distribution, content, and intake. Annu. Rev. Food Sci. T.

[3] Wittstock U., Kurzbach E., Herfurth A.M., Stauber E.J., Kopriva S. (2016). Chapter Six—Glucosinolate Breakdown. Advances in Botanical Research.

[4] Zhang Z., Ober J.A., Kliebenstein D.J. (2006). Gene Controlling the Quantitative Trait Locus EPITHIOSPECIFIER MODIFIER1 Alters Glucosinolate Hydrolysis and Insect Resistance in Arabidopsis. Plant Cell.

[5] Kuchernig J., Backenköhler A., Lübbecke M., Burow M., Wittstock U. (2011). A thiocyanate-forming protein generates multiple products upon allylglucosinolate breakdown in Thlaspi arvense. Phytochemistry.

[6] Salehin M., Li B., Tang M., Katz E., Song L., Ecker J.R., Kliebenstein D.J., Estelle M. (2019). Auxin-sensitive Aux/IAA proteins mediate drought tolerance in Arabidopsis by regulating glucosinolate levels. Nat. Commun..

[7] Chen J., Ullah C., Reichelt M., Beran F., Yang Z.L., Gershenzon J., Hammerbacher A., Vassão D.G. (2020). The phytopathogenic fungus Sclerotinia sclerotiorum detoxifies plant glucosinolate hydrolysis products via an isothiocyanate hydrolase. Nat. Commun..

[8] Bajpai P.K., Reichelt M., Augustine R., Gershenzon J., Bisht N.C. (2019). Heterotic patterns of primary and secondary metabolites in the oilseed crop *Brassica juncea*. Heredity.

[9] Liu D., Zhang C., Zhang J., Xin X., Wu Q. (2022). Dynamics of the glucosinolate–myrosinase system in tuber mustard (*Brassica juncea* var. tumida) during pickling and its relationship with bacterial communities and fermentation characteristics. Food Res. Int..

[10] Sun B., Tian Y., Chen Q., Zhang Y., Luo Y., Wang Y., Li M.Y., Gong R.G., Wang X.R., Zhang F. (2019). Variations in the glucosinolates of the individual edible parts of three stem mustards (*Brassica juncea*). R. Soc. Open Sci..

[11] Yang J., Wang J., Li Z., Li X., He Z., Zhang L., Sha T., Lyu X., Chen S., Gu Y. (2021). Genomic signatures of vegetable and oilseed allopolyploid *Brassica juncea* and genetic loci controlling the accumulation of glucosinolates. Plant Biotechnol. J..

[12] Miao H., Xia C., Yu S., Wang J., Zhao Y., Wang Q. (2023). Enhancing health-promoting isothiocyanates in Chinese kale sprouts via manipulating BoESP. Hortic. Res.-Engl..

[13] Wang J., Shen Y., Sheng X., Yu H., Song M., Wang Q., Gu H. (2024). Unravelling Glucoraphanin and Glucoerucin Metabolism across Broccoli Sprout Development: Insights from Metabolite and Transcriptome Analysis. Plants.

[14] Púčiková V., Rohn S., Hanschen F.S. (2023). Glucosinolate Accumulation and Hydrolysis in Leafy *Brassica* Vegetables Are Influenced by Leaf Age. J. Agric. Food Chem..

[15] Di H., Ma J., Zhang Y., Wei J., Yang J., Ma J., Bian J., Xu J., Huang Z., Tang Y. (2023). Correlations between flavor and glucosinolates and changes in quality-related physiochemical characteristics of Guizhou suancai during the fermentation process. Food Chem..

[16] Wagner M.R., Mitchell-Olds T. (2023). Soil variation among natural habitats alters glucosinolate content in a wild perennial mustard. J. Exp. Bot..

[17] Tandayu E., Borpatragohain P., Mauleon R., Kretzschmar T. (2022). Genome-Wide Association Reveals Trait Loci for Seed Glucosinolate Accumulation in Indian Mustard (*Brassica juncea* L.). Plants.

[18] Xia R., Xu L., Hao J., Zhang L., Wang S., Zhu Z., Yu Y. (2023). Transcriptome Dynamics of *Brassica juncea* Leaves in Response to Omnivorous Beet Armyworm (*Spodoptera exigua*, Hübner). Int. J. Mol. Sci..

[19] Yang J., Li Z., Lian J., Qi G., Shi P., He J., Hu Z., Zhang M. (2020). Brassicaceae transcriptomes reveal convergent evolution of super-accumulation of sinigrin. Commun. Biol..

[20] Bell L., Oloyede O.O., Lignou S., Wagstaff C., Methven L. (2018). Taste and Flavor Perceptions of Glucosinolates, Isothiocyanates, and Related Compounds. Mol. Nutr. Food Res..

[21] Frazie M., Kim M., Ku K. (2017). Health-Promoting Phytochemicals from 11 Mustard Cultivars at Baby Leaf and Mature Stages. Molecules.

[22] Clay N.K., Adio A.M., Denoux C., Jander G., Ausubel F.M. (2009). Glucosinolate metabolites required for an Arabidopsis innate immune response. Science.

[23] Zhang C., Di H., Lin P., Wang Y., Li Z., Lai Y., Li H., Sun B., Zhang F. (2022). Genotypic variation of glucosinolates and their breakdown products in mustard (*Brassica juncea*) seeds. Sci. Hortic..

[24] Chin H.W., Zeng Q., Lindsay R.C. (1996). Occurrence and Flavor Properties of Sinigrin Hydrolysis Products in Fresh Cabbage. J. Food Sci..

[25] Bhattacharya A., Li Y., Wade K.L., Paonessa J.D., Fahey J.W., Zhang Y. (2010). Allyl isothiocyanate-rich mustard seed powder inhibits bladder cancer growth and muscle invasion. Carcinogenesis.

[26] Miao H., Zeng W., Wang J., Zhang F., Sun B., Wang Q. (2021). Improvement of glucosinolates by metabolic engineering in *Brassica* crops. Abiotech.

[27] Mitreiter S., Gigolashvili T. (2021). Regulation of glucosinolate biosynthesis. J. Exp. Bot..

[28] Kliebenstein D.J., Lambrix V.M., Reichelt M., Gershenzon J., Mitchell-Olds T. (2001). Gene duplication in the diversification of secondary metabolism: Tandem 2-oxoglutarate-dependent dioxygenases control glucosinolate biosynthesis in Arabidopsis. Plant Cell.

[29] Kissen R., Bones A.M. (2009). Nitrile-specifier proteins involved in glucosinolate hydrolysis in Arabidopsis thaliana. J. Biol. Chem..

[30] Burow M., Markert J., Gershenzon J., Wittstock U. (2006). Comparative biochemical characterization of nitrile-forming proteins from plants and insects that alter myrosinase-catalysed hydrolysis of glucosinolates. FEBS J..

[31] Matusheski N.V., Swarup R., Juvik J.A., Mithen R., Bennett M., Jeffery E.H. (2006). Epithiospecifier Protein from Broccoli (*Brassica oleracea* L. ssp. *italica*) Inhibits Formation of the Anticancer Agent Sulforaphane. J. Agric. Food Chem..

[32] Kuchernig J.C., Burow M., Wittstock U. (2012). Evolution of specifier proteins in glucosinolate-containing plants. BMC Evol. Biol..

[33] Zeng W., Tao H., Li Y., Wang J., Xia C., Li S., Wang M., Wang Q., Miao H. (2021). The flavor of Chinese kale sprouts is affected by genotypic variation of glucosinolates and their breakdown products. Food Chem..

[34] Chen C., Chen H., Zhang Y., Thomas H.R., Frank M.H., He Y., Xia R. (2020). TBtools: An Integrative Toolkit Developed for Interactive Analyses of Big Biological Data. Mol. Plant.

[35] Tang D., Chen M., Huang X., Zhang G., Zeng L., Zhang G., Wu S., Wang Y. (2023). SRplot: A free online platform for data visualization and graphing. PLoS ONE.

