# Supplementary material for: Integrative Analyses of Metabolites and Transcriptome Reveal the Metabolic Pattern of Glucosinolates in Potherb Mustard (Brassica juncea var. multiceps)
Source: Plants (Basel). 2024 Sep 5;13(17):2481. doi: 10.3390/plants13172481 (PMC11396798; doi:10.3390/plants13172481)
Supplement: Supplementary file 1 [file plants-13-02481-s001.zip › plants-3090326-supplementary.pdf]

Supplementary Table S1 Trivial and chemical name of glucosinolates detected in this study.

Glucosinolate		Trivial name	Abbreviation	Chemical name
Aliphatic glucosinolate	3C	Glucoiberverin	GIV	3-Methylthiopropyl
		Glucoiberin	GIB	3-Methylsulfinylpropyl
		Sinigrin	SIN	2-Propenyl
	4C	Gluconapin	GNA	3-Butenyl
		Progoitrin	PRO	2-Hydroxy-3-butenyl
	5C	Glucobrassicanapin	GBN	4-Pentenyl
Indolic glucosinolate		Glucobrassicin	GBS	3-Indolylmethyl
		Neoglucobrassicin	NGBS	1-Methoxy-3-indolylmethyl
		4-hydroxyglucobrassicin	4-OHGBS	4-Hydroxy-3-indolylmethyl
		4-methoxyglucobrassicin	4-OMGBS	4-Methoxy-3-indolylmethyl
Benzenic glucosinolate		Gluconasturtiin	GST	2-Phenylethyl

Supplementary Table S2 The content of GSL in potherb mustard of different varieties (μmol/g DW).

	**Aliphatics**						**Indoles**				**Benzenics**	**Total**
	**GIB**	**PRO**	**SIN**	**GNA**	**GIV**	**GBN**	**4-OHGBS**	**GBS**	**4-OMGBS**	**NGBS**	**GST**	**glucosinolates**
X1	0.04	0.05	46.29	0.20	0.08	ND	0.49	0.28	0.38	0.09	ND	48.04
X2	0.05	0.09	58.89	0.60	0.12	ND	0.07	0.22	0.27	0.10	ND	60.55
X3	0.07	0.09	69.91	0.82	0.04	0.04	0.30	0.21	0.40	0.09	ND	72.10
X4	0.03	0.04	31.73	0.36	0.14	ND	0.12	0.06	0.17	0.02	ND	32.82
X5	0.03	0.04	38.79	0.45	0.23	ND	0.11	0.07	0.17	0.03	ND	40.09
X6	0.06	0.07	46.27	0.26	0.08	ND	0.17	0.43	0.17	0.10	ND	47.74
X7	0.06	0.08	52.14	0.89	0.62	ND	0.14	0.29	0.29	0.07	ND	54.87
X8	0.04	0.06	33.31	0.23	0.13	ND	0.12	0.67	0.25	0.52	ND	37.07
X9	0.03	0.06	28.77	4.87	0.05	0.37	0.06	0.20	0.30	0.11	ND	34.98
X10	0.04	0.07	40.84	0.57	0.06	ND	0.04	0.49	0.31	0.07	ND	42.67
X11	0.10	0.16	87.06	3.52	0.14	0.09	0.06	0.25	0.23	0.07	ND	91.85
X12	0.01	0.03	29.14	0.12	0.03	ND	0.16	0.43	0.23	0.11	ND	31.72
X13	0.02	0.03	21.81	0.12	0.01	ND	ND	0.44	0.22	0.06	ND	24.25
X14	0.06	0.05	22.61	0.10	0.09	ND	0.05	0.42	0.32	0.11	ND	25.66
X15	0.03	0.06	56.92	0.41	0.25	ND	ND	0.22	0.11	0.09	0.70	60.60
X16	0.03	0.02	30.40	1.20	ND	0.02	0.02	0.03	0.18	0.03	ND	32.12
X17	0.05	0.06	39.28	0.21	0.15	ND	ND	0.33	0.15	0.06	0.18	40.78
X18	0.06	0.09	59.03	0.38	0.25	ND	0.08	0.44	0.27	0.19	ND	60.94
X19	0.06	0.06	45.94	0.62	0.09	ND	0.09	0.37	0.28	0.36	ND	49.31
X20	0.05	0.06	35.32	0.49	0.13	ND	ND	0.21	0.10	0.02	ND	37.95
X21	0.04	0.04	24.10	0.85	0.03	ND	0.03	0.07	0.04	0.02	ND	25.36
X22	0.06	0.06	41.11	0.63	0.47	ND	0.10	0.28	0.28	0.06	ND	43.32

X23	0.03	0.05	38.17	0.67	0.02	0.04	0.07	0.09	0.10	0.06	ND	39.40
X24	0.09	0.09	41.92	0.26	0.08	ND	0.06	0.32	0.29	0.42	ND	43.74
X25	0.04	0.05	50.31	0.28	ND	ND	0.15	0.19	0.28	0.15	ND	51.72
X26	0.04	0.04	41.90	0.31	0.02	ND	ND	0.10	0.07	0.05	0.08	44.18
X27	0.07	0.07	32.59	0.40	0.06	ND	0.02	0.19	0.15	0.05	ND	35.35
X28	0.09	0.07	33.98	0.53	0.10	ND	ND	0.34	0.22	0.13	ND	37.03
X29	0.09	0.11	49.72	0.79	0.14	ND	ND	0.28	0.22	0.11	0.11	53.30
X30	0.06	0.11	50.79	0.33	0.09	ND	0.19	0.20	0.27	0.22	0.13	54.04
X31	0.04	0.07	49.60	0.72	0.07	ND	0.02	0.30	0.41	0.08	ND	53.02
X32	0.06	0.04	31.58	0.27	0.11	ND	ND	0.09	0.05	0.01	0.09	34.02
X33	0.05	0.05	58.28	0.28	ND	ND	ND	0.13	0.09	0.05	ND	60.47
X34	0.05	0.04	37.34	0.15	ND	ND	0.01	0.32	0.21	0.03	ND	38.45
X35	0.02	0.02	18.98	1.07	ND	ND	ND	0.03	0.02	0.02	ND	22.11
X36	0.02	0.05	27.08	5.15	ND	0.41	ND	0.03	0.08	0.05	ND	33.21
X37	0.03	0.05	34.33	0.16	0.03	ND	ND	0.55	0.19	0.17	ND	35.80
X38	0.06	0.12	46.38	6.93	ND	0.42	0.05	1.70	0.20	0.15	ND	56.40
X39	0.03	0.05	32.30	0.22	0.11	ND	0.01	0.45	0.11	0.19	ND	35.14
X40	ND	0.07	40.84	0.41	ND	ND	ND	0.45	0.10	0.27	0.18	42.67
X41	0.02	0.04	33.10	0.09	0.07	ND	ND	0.25	0.21	0.06	ND	34.17
X42	0.05	0.12	64.59	1.51	0.10	0.02	ND	0.25	0.19	0.05	0.13	68.75
X43	0.34	0.10	46.86	0.31	1.45	ND	0.03	0.11	0.39	0.10	ND	49.89
X44	0.02	0.08	26.05	0.11	0.04	ND	ND	0.05	0.07	0.06	0.20	27.01
X45	0.02	0.05	28.86	0.13	0.06	ND	ND	0.16	0.40	0.08	ND	29.75
X46	0.03	0.05	55.98	0.24	0.07	ND	ND	0.50	0.32	0.05	ND	57.58
X47	ND	0.02	15.22	0.14	0.11	ND	0.01	0.13	0.10	0.02	ND	17.46
X48	0.60	0.08	24.18	0.32	0.03	ND	ND	0.14	0.13	0.03	ND	27.23

X49	ND	0.07	53.06	0.85	0.15	0.03	ND	0.39	0.22	0.03	0.12	56.61
X50	0.05	0.06	29.29	2.36	ND	0.14	ND	0.26	0.10	0.02	ND	32.55
X51	0.04	0.04	34.44	0.60	0.06	0.05	0.01	0.08	0.05	0.03	ND	35.51
X52	0.05	0.05	25.48	0.28	0.04	ND	ND	0.21	0.02	0.03	0.06	27.94
X53	0.07	0.06	34.90	0.85	ND	ND	ND	0.17	0.05	0.04	0.03	37.78
X54	0.11	0.08	39.69	0.35	0.02	ND	0.03	0.31	0.16	0.02	0.09	42.61
X55	0.05	0.07	45.60	2.60	0.07	0.16	ND	0.27	0.03	0.02	0.03	50.52
X56	0.03	0.03	25.45	0.12	0.05	ND	ND	0.15	0.04	0.01	0.03	27.69
X57	0.06	0.05	23.38	1.77	0.02	0.10	ND	0.22	0.03	0.01	0.02	27.28
X58	0.08	0.08	38.58	0.25	0.01	ND	ND	0.15	0.01	0.02	0.05	41.03
X59	0.09	0.05	28.06	0.23	ND	ND	ND	0.20	0.01	0.01	0.04	30.30
X60	0.04	0.04	25.44	2.47	0.03	0.10	ND	0.20	0.03	0.02	0.01	30.17
X61	0.12	0.08	41.47	0.43	ND	ND	ND	0.18	0.06	0.01	0.12	44.24
X62	0.06	0.05	26.79	0.32	ND	ND	ND	0.19	0.03	0.01	0.03	29.07
X63	0.03	0.04	35.23	0.56	0.18	ND	ND	0.12	0.12	0.05	ND	37.98
X64	0.06	0.09	51.81	4.88	ND	0.19	0.02	0.27	0.15	0.05	0.15	59.32
X65	0.23	0.07	30.99	3.67	ND	0.22	ND	0.14	0.09	0.02	0.07	37.31
X66	0.06	0.06	43.19	1.29	0.04	ND	ND	0.17	0.22	0.01	ND	46.77
X67	0.03	0.03	22.83	0.34	ND	ND	ND	0.05	0.06	0.01	ND	25.03
X68	0.04	0.08	43.93	0.69	0.14	ND	0.01	0.24	0.39	0.15	ND	47.24

GBN, glucobrassicanapin; GIB, glucoiberin; SIN, sinigrin; GNA, gluconapin; PRO, progoitrin; GIV, glucoiberverin; 4-OHGBS, 4-hydroxyglucobrassicin; GBS, glucobrassicin; 4-OMGBS, 4-methoxyglucobrassicin; NGBS, neoglucobrassicin; GST, gluconasturtiin; Aliphatics, aliphatic glucosinolates; Indoles, indolic glucosinolates; Benzenics, Benzenic glucosinolates. ND, not detected.

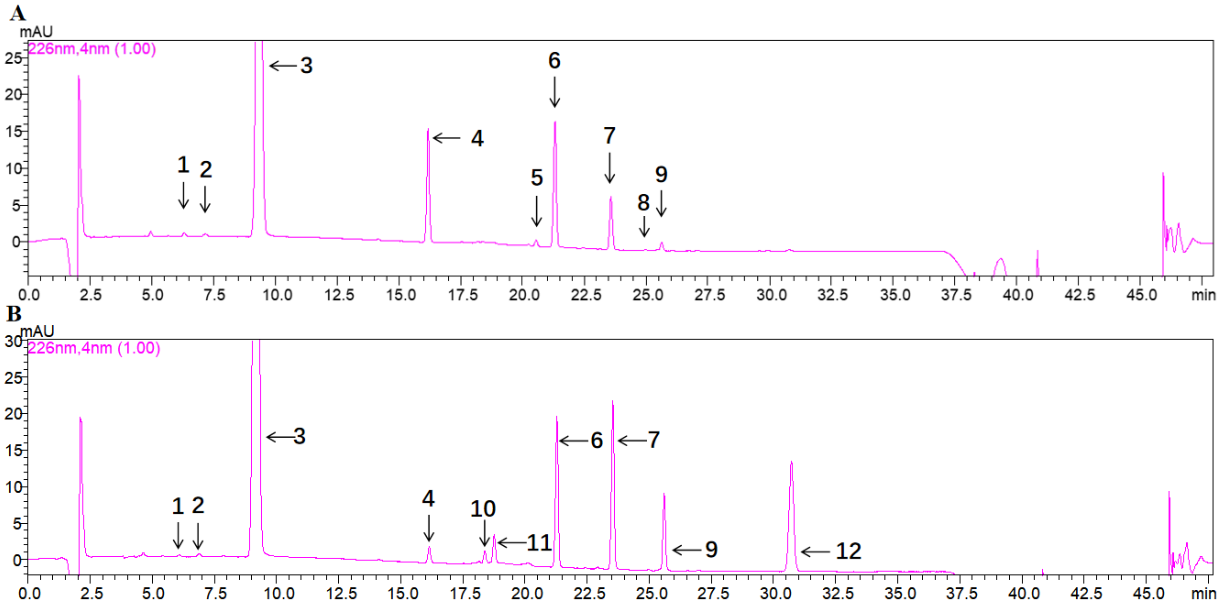

Supplementary Figure S1 HPLC chromatograms of desulfoglucosinolates prepared from potherb mustard. 1: GIB, Glucoiberin; 2: PRO, Progoitrin; 3: SIN, Sinigrin; 4: GNA, Gluconapin; 5: ONPG, Ortho-nitrophenyl-β-D-galactopyranoside (internal standard); 6: GBN, Glucobrassicanapin; 7: GBS, Glucobrassicin; 8: GST, Gluconasturtiin; 9: 4-OMGBS, 4-Methoxyglucobrassicin; 10: GIV, Glucoibervirin; 11: 4-OHGBS, 4-Hydroxy-3-indolylmethyl GSL; 12: NGBS, Neoglucobrassicin.

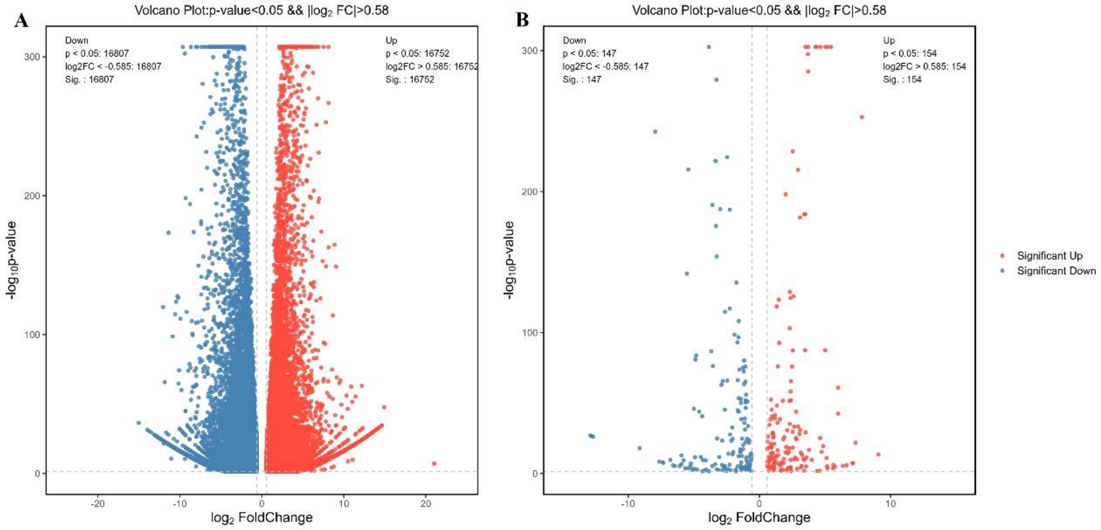

Supplementary Figure S2 Volcano plot of all the differentially expressed genes (A) and glucosinolate metabolism-related differentially expressed genes (B) in X57 vs X11

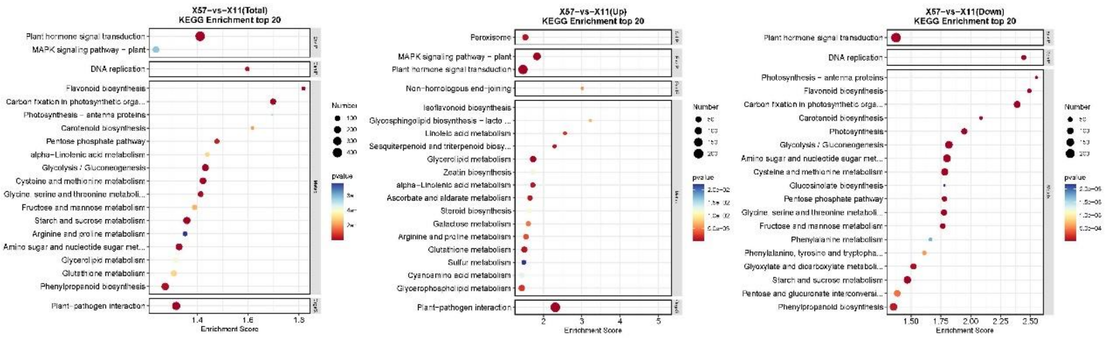

Supplementary Figure S3 KEGG enrichment analysis of differentially expressed genes in X57 vs X11.

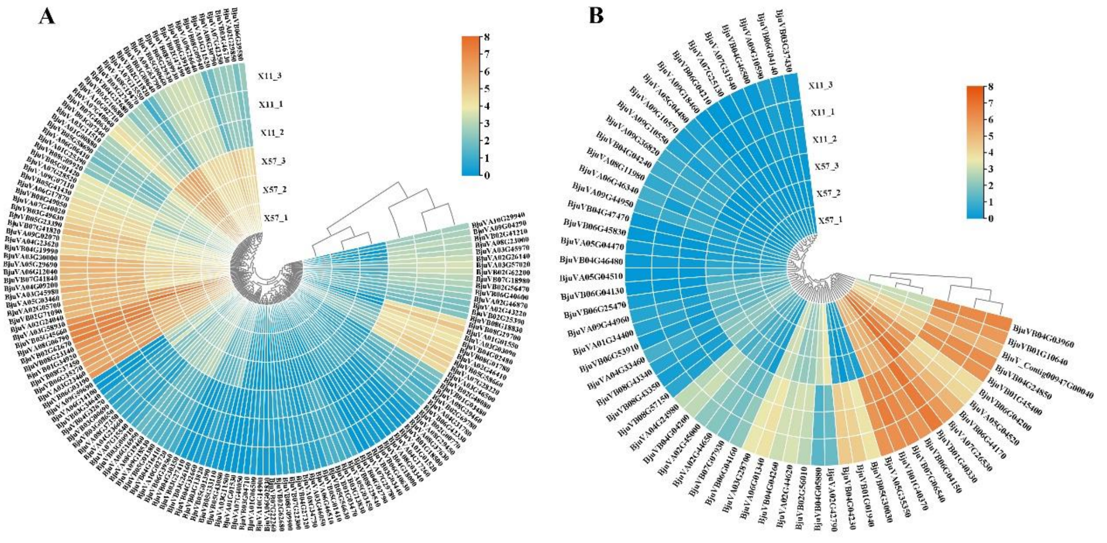

Supplementary Figure S4 The changes of differentially expressed genes related to glucosinolate biosynthesis (A) and degradation (B) in X57 and X11. Values represent the means of three replicates. The expression levels were visualized using TBtools.
